# Optimizing Precision Oncology: Structural Frameworks for Local MTB Integration and Outcome Assessment

**DOI:** 10.3390/medsci14020242

**Published:** 2026-05-07

**Authors:** Nicoleta Zenovia Antone, Flaviu Andreicovici, Andrada Larisa Deac, Roxana Pintican, Maria Miclaus, Adrian Pavel Trifa, Andreea Catana, Ovidiu Balacescu, Cătălin Vlad, Patriciu Achimas-Cadariu

**Affiliations:** 1 “Iuliu Hatieganu” University of Medicine and Pharmacy, 400012 Cluj-Napoca, Romania; 2 Breast Cancer Center, Institute of Oncology “Prof. Dr. Ion Chiricuta”, 400015 Cluj-Napoca, Romania; 3 Department of Medical Oncology, Emergency Hospital Cluj-Napoca, 400535 Cluj-Napoca, Romania; 4 Department of Radiology, “Iuliu Hatieganu” University of Medicine and Pharmacy, 400012 Cluj-Napoca, Romania; 5 Department of Genetic Explorations, “Prof. Dr. Ion Chiricuta” Institute of Oncology, 400015 Cluj-Napoca, Romania; 6 Center for Research and Innovation in Personalized Medicine of Respiratory Diseases, Discipline of Medical Genetics, “Victor Babes” University of Medicine and Pharmacy, 300041 Timisoara, Romania; 7 Center of Expertise on Rare Pulmonary Diseases, Clinical Hospital of Infectious Diseases and Pneumophysiology “Dr. Victor Babes”, 300310 Timisoara, Romania; 8 Department of Molecular Sciences, “Iuliu Hatieganu” University of Medicine and Pharmacy, 400012 Cluj-Napoca, Romania; 9 Department of Surgery, Institute of Oncology “Prof. Dr. Ion Chiricuta”, 400015 Cluj-Napoca, Romania

**Keywords:** precision oncology, molecular tumor board, best practices

## Abstract

Background/Objectives: Molecular tumor profiling has recently transformed oncologic care delivery, establishing precision medicine as an essential approach for defining cancer biology and revealing intratumoral heterogeneity. The growing accessibility of advanced nucleic acid sequencing technologies has created a demand for specialized expertise in interpreting comprehensive genomic profiling results. Academic institutions currently employ a strategy of conducting initial broad-spectrum genomic testing, followed by matching patients to investigational therapies targeting their specific genomic alterations. Consequently, molecular tumor boards (MTBs) have emerged predominantly within major cancer centers and academic medical institutions, providing the specialized knowledge necessary to translate precision oncology into routine clinical care. However, despite the substantial benefits of collaborative case review within tumor boards, clinicians frequently encounter multiple barriers to effective MTB implementation. Methods: this report examines these challenges performing an exploratory quantitative synthesis approach and explores implementation strategies and best practices derived from collective institutional experiences, with the goal of establishing a functional MTB at the local level and thereby expanding oncology patient access to cutting-edge therapeutic options.

## 1. Introduction

Molecular oncology has experienced significant advances through breakthrough discoveries in genetic testing and the expanding clinical implementation of comprehensive genomic profiling via next-generation sequencing (NGS) [1,2,3,4,5,6]. These technologies enable precise molecular characterization of cancer biology and identification of potentially actionable alterations [1]. Despite lacking health insurance reimbursement in most countries, gene panels have become progressively more accessible due to steadily decreasing costs, while simultaneously expanding in complexity regarding the spectrum of mutations evaluated [3,4]. These developments have transformed oncological care delivery, with precision oncology now integrated into clinical practice and contemporary treatment guidelines, driving a paradigm shift from conventional chemotherapy-based regimens toward individualized, molecularly guided targeted therapies [3,5,6,7].

The expanding volume of tumor genomic data and growing knowledge of molecular alterations in oncogenic pathways have created demand for specialized expertise in this oncology subspecialty [8,9]. The increasing complexity of cancer management has promoted multidisciplinary collaboration and multimodal treatment approaches designed to optimize therapeutic strategies and achieve the best possible patient outcomes [3,6,10]. Molecular tumor boards have emerged as valuable resources for medical oncologists confronting substantial genomic data from comprehensive testing that requires expert interpretation and clinical translation, despite limited formal training in molecular oncology [11,12,13].

A molecular tumor board is a multidisciplinary, histology-agnostic forum that convenes diverse specialists who, unlike conventional tumor boards, prioritize molecular characteristics over anatomic location and histopathology, thereby operationalizing precision oncology through expert translation of genetic panel data into targeted therapy recommendations and optimal cancer care across all available treatment modalities [7,14,15].

The essential MTB members are:

- Medical oncologist(s) with training in cancer genomics, with a role in assessing the relationships of molecular abnormalities to cancer prognosis and therapy response;
- Molecular pathologist(s), with a role in elaborating on tumor histology and other biomarkers that could be further assessed;
- Molecular biologists, with a role in interpreting the functional relevance of molecular abnormalities;
- Clinical geneticists/genomic counselors, with a role in elucidating germline aberrations and experimental treatment options and providing in-depth knowledge of cancer-related pathways and interpret genomic variants;
- Representatives of the clinical care team for each patient.

Optional participants include bioinformatics, MTB administrators, pharmacists, patient coordinators, residents, or clinical trialists. Also, the MTB should be described as part of or separate from the clinical multidisciplinary tumor board in the institution [1,2,3].

Cases are typically referred to MTBs in situations where standard treatment pathways are insufficient to guide management. Common indications include rare, complex or atypical tumor biology, progression after standard therapy, discordance between histology and molecular profile, presence of potentially actionable genomic alterations, consideration of off-label targeted therapy, or suspicion of hereditary cancer predisposition. Before discussion, the case must be verified for: completeness of clinical information, adequate molecular testing, technical quality of sequencing and clear clinical objective [1,2,4].

Advances in precision oncology have established tumor genomic profiling as a biomarker for therapeutic decision-making, now routinely utilized in first-line treatment selection and increasingly applied in adjuvant and neoadjuvant settings [3,10,16]. Despite demonstrated benefits and improved outcomes—including enhanced clinical benefit, progression-free survival, and overall survival with adherence to MTB recommendations—precision medicine remains underutilized, particularly in developing countries, rural regions, and small community oncology centers where tumor board access is limited and human or financial resources are constrained [4,7,11,12,17,18].

This literature narrative review primarily aims to identify and synthesize best practices for molecular tumor boards from available published data shared by leading oncological institutions, explore implementation strategies, and develop a reproducible framework adaptable for establishing a molecular tumor board at local facilities.

Our secondary objectives are to summarize patient outcomes (progression-free survival and overall survival) among cases reviewed by molecular tumor boards and characterize the genomic landscape identified through comprehensive genomic profiling.

## 2. Materials and Methods

Molecular tumor boards are regularly scheduled meetings held at select cancer centers, convened at institution-specific intervals in either physical or hybrid formats. These forums assemble multidisciplinary teams with expertise spanning multiple domains of cancer care, typically including specialists with advanced training in medical and radiation oncology, surgery, clinical genetics, pathology, radiology, clinical pharmacy, translational research, and tumor biology. Under the leadership of a senior physician, these interdisciplinary teams collaborate to formulate optimal treatment strategies for complex or rare cases based on genetic testing data [3,4,6,10,11,12,19,20].

A narrative literature search was performed between June 2014 and May 2024 using the PubMed electronic database with the search terms ‘Molecular Tumor Board (title)’ and ‘English (language)’, yielding 62 articles. Title and abstract screening led to the exclusion of 14 articles that did not align with the study objectives of providing implementation strategies and describing associated challenges for establishing an MTB in a local oncology center.

Specifically, case reports and studies focused on pediatric populations were excluded. The final review included 48 articles published from June 2014 to May 2023.

Descriptive variables were summarized as counts and percentages. For study-level categorical outcomes, proportions were calculated, and key findings were reported as frequencies across included studies.

Given the heterogeneity of study designs, tumor types, and reporting formats, a formal inverse-variance meta-analysis was not feasible for all endpoints. Therefore, an exploratory quantitative synthesis approach was applied.

For time-to-event outcomes, when hazard ratios (HRs) were directly reported, cross-study effect sizes were summarized using the geometric mean of the reported HRs. When studies reported only median progression-free survival (PFS) or overall survival (OS), cross-study comparisons were summarized using ratios of median survival between MTB-guided and comparator groups, and geometric means of these ratios were calculated.

Additionally, ranges and distributions of reported survival outcomes were summarized across studies. Where available, interquartile ranges (IQRs) were reported descriptively.

For genomic alterations, a study-level occurrence approach was used, whereby the frequency of each mutation was calculated based on the number of studies reporting that alteration, rather than patient-level prevalence.

Due to incomplete reporting of variance measures, sample sizes, and confidence intervals across studies, pooled estimates should be interpreted as exploratory and hypothesis-generating rather than definitive. Statistical heterogeneity was assessed qualitatively based on differences in tumor type, study population, MTB structure, and comparator definitions.

In this review, the literature search was limited to PubMed, as this database provides near-complete coverage of studies relevant to molecular tumor boards (MTBs) within clinical oncology and precision medicine. Preliminary scoping checks performed in Embase and Scopus indicated that almost all MTB-related records retrieved from these sources were duplicates of PubMed entries, with only rare additional studies that were outside the scope of clinical implementation research. Because MTB terminology is highly specific and standardized (e.g., “molecular tumor board”, “molecular tumour board”, “MTB”), the likelihood of unique records appearing exclusively in other databases is low. Given the minimal incremental yield and the substantial overlap with PubMed, expanding the search to multiple databases was not methodologically justified. To strengthen comprehensiveness, the PubMed strategy was refined to include both keyword and abstract searches, and the rationale for database selection is explicitly detailed here.

## 3. Results

Optimal meeting frequency varies depending on the institutional caseload. Among reviewed articles, six reported weekly MTB meetings [5,9,13,21,22,23], four described a format of three meetings monthly [12,18,24,25], seven documented biweekly sessions [3,4,6,11,17,26,27], and three indicated monthly scheduling [8,9,10]. Notably, meeting frequency may evolve based on demand: Trivedi et al. transitioned from biweekly to monthly meetings [3], whereas Heinrich et al. increased from bimonthly to weekly sessions due to rising case volumes [26]. Beyond standard weekly local MTBs, Jain et al. reported that one center established a supplementary monthly virtual tumor board to extend access to smaller community practices [9]. The virtual format serves as an alternative when in-person meetings are impractical due to physician scheduling constraints, epidemiologic circumstances, or when academic centers provide MTB access to affiliated oncology practices in a time- and location-independent manner, as documented in six reviewed articles [9,11,17,20,26,28].

**Figure 1:**
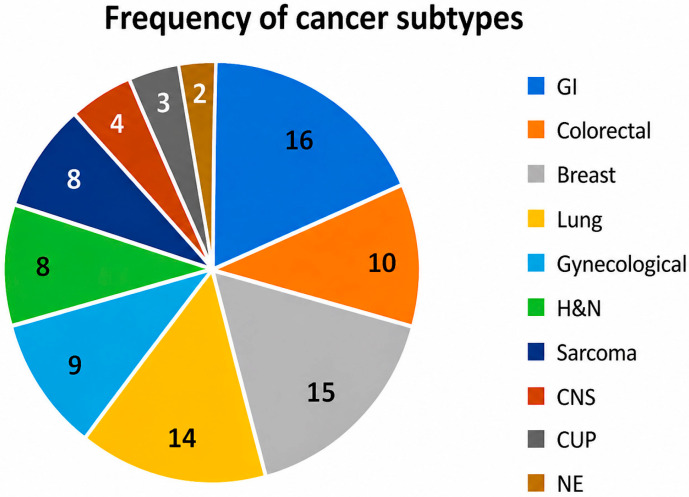
Frequency of cancer subtypes (Legend: GI = Gastrointestinal, H&N = Head and neck, CNS = Central nervous system, CUP = Cancer of unknown primary, NE = Neuroendocrine).

Performing an exploratory quantitative synthesis among the 48 reviewed articles, gastrointestinal cancers were most frequently represented, appearing in 16 studies [3,4,5,6,7,8,9,11,16,18,23,24,26,28,29,30], with colorectal cancer specifically addressed in 10 [3,5,6,8,9,18,23,24,26,30]. Breast cancer followed in 15 studies [3,4,6,8,9,10,11,12,15,18,23,25,26,28,30], lung cancer in 14 [3,4,7,8,9,13,15,17,18,23,26,28,31,32], gynecological malignancies in nine [3,5,7,10,12,18,27,28,30], and head and neck cancers and sarcomas in eight articles each (Figure 1) [5,8,14,16,18,23,28,30]. Less commonly reported were central nervous system tumors (four articles) [8,10,14,23], cancers of unknown primary (three articles) [8,14,23], and neuroendocrine tumors (two articles) [16,18].

Genetic profiling constitutes the cornerstone of tumor boards, enabling the delivery of precision oncology [4,14]. The expanding availability of comprehensive genomic profiling facilitates establishment of a genomic landscape [2,16,18]. Among the 48 reviewed papers, 14 articles reported the frequency of genetic alterations identified through NGS tissue-based testing (Table 1) [4,5,7,8,10,12,14,15,18,24,25,26,29,33]. Given the substantial number of alterations documented in individual studies, up to seven of the most prevalent genetic alterations were reported for each study. Frequency of genetic alterations for NGS tissue-based testing reported for specific tumor subtypes are reported in Table 2. 

TP53 mutations were identified across fourteen articles [4,5,7,8,10,12,14,15,18,24,25,26,29,33]. KRAS and PIK3CA gene mutations were each reported in eleven studies [4,5,8,10,12,14,15,18,24,25,26,29,33]. CDKN2A/B alterations appeared in seven articles, as did mutations in APC and MYC genes [4,5,7,8,14,15,18,24,25,29,33]. ERBB2 gene alterations were documented in six studies, while PTEN mutations were reported in five [4,5,7,8,10,12,14,15,18,25]. BRCA1/2 alterations appeared in three articles, and CCND1 alterations and KIT mutations were each evidenced in two papers [8,10,12,14,25,33]. Single-article reports included mutations in BRAF, ATM, SMAD4, GATA, ARID1A, and EGFR genes [7,10,14,25].

Despite widespread availability of genomic profiling, optimal testing timing remains debated, with existing studies demonstrating both advantages and limitations for early versus late testing; therefore, achieving appropriate balance is essential to ensure favorable clinical impact [3,6,9,10,12,16]. Evidence from several studies suggests that earlier testing during the disease course may be preferable to genomic profiling in advanced disease settings where clinical benefit is unlikely to be significant. However, re-biopsy with additional testing may be necessary in some cases at progression, as cancer biology undergoes substantial changes and archived tissue may no longer be representative [3,4,9,10,15,16].

Circulating tumor DNA (ctDNA) testing offers a suitable alternative to tissue-based analysis, providing valuable information on acquired resistance mutations, clonal dynamics, and tumor heterogeneity at any point during disease evolution and at reduced cost, thereby circumventing the challenge of optimal testing timing [15,24,34,35]. Although liquid biopsy was utilized significantly less frequently than tissue-based testing for genomic profiling in the reviewed studies, the information obtained remains highly valuable. Three articles reported mutation profiles derived from ctDNA analysis, with TP53 mutation identified as the most frequent alteration across all three studies, followed by KRAS, PIK3CA, and BRAF mutations, as well as MYC amplification reported in two studies each [12,18,24]. These findings support ctDNA as a viable, reproducible, and cost-effective testing option.

**Table 1:** Frequency of genetic alterations for NGS tissue-based testing reported for all tumor subtypes.

Article	Frequency of Mutations
Charo et al. [16]	TP53, PIK3CA, MYC, CCND1, PTEN
Dalton et al. [19]	TP53, KRAS, PIK3CA, CDKN2A, MYC, PTEN, ERBB2
Dorman et al. [32]	TP53, KRAS, CDKN2A
Harada et al. [14]	TP53, KRAS, PIK3CA, BRAF, BRCA, ATM, PTEN
Heinrich et al. [29]	TP53, KRAS
Hoefflin et al. [18]	TP53, APC, ATM, SMAD4, ERBB2, PIK3CA, KIT
Hoefflin et al. [36]	TP53, BRCA1/2, KIT, PIK3CA, ATM, KRAS, APC
Kato et al. [22]	TP53, KRAS, PIK3CA, CDKN2A/B, APC, MYC, ERBB2
Louie et al. [27]	TP53, KRAS, PIK3CA, APC, SMAD4, MYC, FLT3
Miller et al. [11]	TP53, CDKN2A/B, ERBB2, EGFR, KRAS, ARID1A, NF1
Parker et al. [28]	TP53, PIK3CA, MYC, ERBB2, GATA, CCND1, PIK3CA
Schwaederle et al. [8]	TP53, KRAS, PIK3CA, CDKN2A, APC, MYC, PTEN
Shirota et al. [9]	TP53, KRAS, CDKN2A, APC, PIK3CA, BRCA1/2, ERBB2
Taffe et al. [12]	TP53, KRAS, PIK3CA, CDKN2A, APC, MYC, PTEN

**Table 2:** Frequency of genetic alterations for NGS tissue-based testing reported for specific tumor subtypes.

Tumor Type	Article	Frequency of Mutations
Breast	Parker et al. [28]	TP53, PIK3CA, MYC, ERBB2, GATA, CCND1
Colo-rectal	Louie et al. [27]	TP53, KRAS, PIK3CA, APC, SMAD4, MYC, FLT3
GI * (Pancreas)	Dorman et al. [32]	TP53, KRAS, CDKN2A

* GI = Gastro-intestinal.

Selection of optimal testing panels represents another important consideration. A study by Tarawneh et al. suggests that focused panel sequencing may be more sensitive at early disease stages for certain cancer types, yielding comparable results at reduced cost with faster turnaround and more straightforward interpretation [16]. Conversely, comprehensive gene panels may benefit patients with advanced disease and limited treatment options, despite greater financial burden and time-intensive interpretation, by identifying potentially actionable mutations that could provide clinical trial eligibility or access to investigational agents. An additional advantage of MTB discussion is the capacity to provide recommendations for incidental findings, including germline mutations or variants of unknown significance (VUS), enabling personalized surveillance and timely detection and intervention for patients and their families [8,9,34].

Turnaround time—the interval from biopsy collection to MTB recommendation—warrants particular attention and demonstrates considerable variability across studies. Available data indicates an approximate 28-day interval in three articles, while another study reported an improved turnaround time of approximately 14 days attributable to automated data interpretation process [1,10,14,33].

Among the 48 articles analyzed, 18 reported rates of actionable mutations identified through genetic testing or rates of MTB recommendations based on actionable alterations discovered via comprehensive genomic profiling. Eight studies documented rates exceeding 70% [3,11,13,15,16,25,28,31], while the remaining studies reported rates ranging from 41% to 69% [5,8,12,14,18,24,26,29,30,36]. The significance of genetic testing is underscored by these high rates of positive results for actionable mutations or issued recommendations, with some articles reporting identification of at least one actionable alteration in up to 93% of patients tested [3,5,8,11,13,25,28,30,31,36]. These findings emphasize the need to improve patient access to genetic testing and encourage physicians to order genomic profiling when available.

1. Quantitative synthesis of survival outcomes

Across the included studies, molecular tumor board (MTB)-guided management was consistently associated with improved survival outcomes. Among studies reporting hazard ratios (HRs) for progression-free survival (PFS), the reported values were 0.50 (Charo et al. [16]), 0.63 (Kato et al. [22]), 0.48 (Louie et al. [27]), and 0.41 (Louie et al. [27], colorectal cancer subgroup). An exploratory cross-study synthesis using the geometric mean yielded a pooled HR of approximately 0.50, indicating a consistent reduction in the risk of progression associated with MTB-guided treatment. Similarly, for overall survival (OS), the reported HRs were 0.64 (Charo et al. [16]), 0.67 (Kato et al. [22]), and 0.46 (Louie et al. [27]), corresponding to an exploratory geometric mean HR of approximately 0.58, suggesting a favorable survival benefit in patients managed following MTB recommendations.

In addition to HR-based analyses, several studies reported median survival values. Across these studies, the geometric mean ratio of MTB-guided versus comparator median PFS values was approximately 1.91, while the corresponding ratio for OS was approximately 2.11, further supporting a clinically meaningful survival advantage associated with MTB-guided care.

2. Distribution of survival outcomes

Across studies reporting median PFS, MTB-guided treatment was associated with median PFS values ranging from 4.3 to 9.3 months, compared to 1.9 to 4 months in comparator groups (Table 3 and Table 4). For OS, reported median values ranged from 15.3 to 18 months in MTB-guided cohorts versus 4.7 to 10.8 months in comparator groups (Table 3 and Table 4). In studies reporting survival distributions, interquartile ranges (IQR) further highlighted variability across populations, with one study reporting a median PFS of 6.3 months (IQR 3.2–10.6) and OS of 10.4 months (IQR 6.3–14.6), reflecting heterogeneity in clinical outcomes across tumor types and treatment settings (Table 5).

3. Frequency of genetic alterations—quantitative summary

A total of 14 studies reported frequencies of genetic alterations identified through tissue-based next-generation sequencing. Using a study-level occurrence approach, TP53 mutations were identified in 100% of studies (14/14), followed by KRAS and PIK3CA mutations, each present in 78.6% of studies (11/14). CDKN2A/B, APC, and MYC alterations were reported in approximately 50% of studies, while ERBB2 and PTEN alterations were less frequently observed. This distribution highlights TP53 as the most consistently reported genomic alteration across tumor types, followed by recurrent alterations in key oncogenic signaling pathways.

4. Actionable alterations and MTB recommendations

Among the studies reporting actionable mutation rates or MTB recommendations, eight studies documented rates exceeding 70%, while the remaining studies reported rates ranging from 41% to 69%. Across these studies, the median reported rate of actionable alterations or MTB recommendations was approximately 65–70%, indicating a high likelihood of identifying clinically relevant targets through comprehensive genomic profiling. However, implementation of MTB recommendations remained limited, with most studies reporting adherence rates below 40%, despite evidence of improved clinical outcome.

Integrating MTBs into routine clinical practice confers substantial benefit for both patients and physicians [17,18]. The majority of available data associate MTB implementation with clinical benefit and positive outcomes, suggesting improvements in progression-free survival (PFS), overall survival (OS), and response rates (RR) when tumor board recommendations are followed. Seven articles documented PFS benefit and seven reported OS benefit when cases underwent multidisciplinary review and recommendations were implemented in clinical practice (Table 2), whereas four papers reported no OS benefit and one reported no PFS benefit [7,12,14,15,16,17,18,24,25,30,32,33,34]. Six articles described additional significant clinical benefit when MTB recommendations were followed [4,11,15,24,25,33].

Although treatment recommendations were provided in most cases, the decision to implement MTB guidance remained at the discretion of the treating physician, who elected to follow recommended treatment in a limited proportion of cases—typically below 40%—as reported in eight articles. This low adherence rate persists despite demonstrated improved outcomes and reflects limited access to clinical trials and targeted therapies [3,11,14,16,25,26,29,31]. Only two studies reported high adherence rates of 81% and 86% [28,32].

According to most research, MTB discussions facilitate easier access to experimental drugs for patients with actionable mutations. However, even though clinical trials are highly recommended, with some studies showing a recommendation rate as high as 83% [15], the actual enrollment rate remains low [6,11,14,15,19,25,26,28]. This issue is largely due to strict eligibility criteria, patients declining participation because of financial constraints or the distance required to travel for the trial, physicians’ hesitance, patients’ performance status, or the lack of targetable mutations [6,11,14,15,18,25,28].

Significant concerns in this context are represented by stringent eligibility criteria, patients’ refusal due to financial burdens or the distance required to travel for clinical trials, physicians’ reluctance, performance status, or the absence of targetable mutations [6,11,14,15,18,25,28]. A potential strategy to enhance trial inclusion and drug availability in rural areas and developing countries is to facilitate access for small community hospitals to expertise from major cancer centers through virtual tumor boards, as described in the study by Jain et al. [9]. Therefore, the implementation of MTBs in daily clinical practice is essential, as it leads to the standardization of recommendations and treatments, thereby increasing the availability of current therapeutic options for patients with advanced or metastatic disease whose options are limited, based on genomic profiling [3,19,35,37].

## 4. Discussion

The present review highlights the growing role of molecular tumor boards (MTBs) in translating genomic data into clinically actionable treatment strategies. The exploratory quantitative synthesis provides additional insight into the magnitude and consistency of clinical benefit associated with MTB-guided care.

MTBs, typically implemented in academic cancer centers, represent an evolution of the traditional multidisciplinary tumor board, now widely adopted in oncology practice [17,38,39,40,41,42]. In resource-limited settings, adapted formats such as mini tumor boards may support personalized treatment while improving adherence to guidelines and optimizing resource allocation [3,4,6,11,19,21,43]. Despite implementation challenges, MTBs contribute to improved care organization, interdisciplinary collaboration, and integration of evidence-based recommendations [9,12,14,16,18,38,44].

As cancer care becomes increasingly complex, the need for specialized teams capable of interpreting genomic data continues to grow. MTBs support clinicians by facilitating consensus and providing expert recommendations in complex cases [3,4,10,19]. This collaborative approach may reduce physician burden, improve confidence in molecular profiling, and support integration of precision oncology into routine practice [3,8,14,20,25]. The case prioritization should focus on cases which impact patient care through clarification of diagnostic findings, prompt additional diagnostic testing or suggest investigational or off-label therapies. Also, MTBs should prioritize treatment options if more than one exists. Furthermore, the focus should be on cases with complex genomic profiles, poorly annotated genomic findings or putative biomarkers of resistance. Also, rare cancers and cancers with limited treatment options should be prioritized [3,4,10,19].

Across studies reporting hazard ratios, MTB-guided management was associated with improved outcomes, including reduced risk of disease progression and death. The pooled estimates suggest approximately a 50% reduction in progression risk and improved overall survival, supported by median-based analyses demonstrating clinically meaningful benefits across heterogeneous populations. These results need to be carefully interpreted as this is an exploratory quantitative synthesis. The geometric mean hazard ratios and median survival ratios are descriptive only and not formal meta-analytic estimates due to the high heterogeneity of the included studies and the lack of consistent variance measures and patient-level data.

MTBs also provide educational value by promoting continuous learning and improving interdisciplinary communication [4,10,11,19,41,44]. However, barriers remain, including limited resources, time constraints, hierarchical dynamics, and lack of standardized frameworks [3,6,17,18,45]. Strategies such as financial incentives and continuing education credits may improve participation [9,40].

MTB recommendations are directly dependent on the quality and completeness of submitted clinical data. Incomplete clinical context may lead to inappropriate therapy selection, whereas excessive unstructured data prevents clear decision-making. The objective of case presentation is not to summarize the entire disease history, but to provide a concise clinical scenario that allows interpretation of molecular findings and selection of a therapeutic strategy [3,4,10,19].

Operational efficiency is also critical. Structured case submission, including clinical and genomic data, enables effective multidisciplinary review and formulation of recommendations [3,7,10,11,12,19,46,47]. These recommendations remain advisory, with final decisions made by the treating physician [3,4,5,7,10,11,12,19]. Formal MTB reports may facilitate access to off-label therapies and support reimbursement decisions [3,8,9,20,37].

Furthermore, the role of MTBs is particularly critical in (ultra)rare and molecularly complex tumors, where genomic alterations are highly context-dependent and their clinical significance often varies by tumor type. In these settings, accurate interpretation frequently requires specialized expertise and coordinated multidisciplinary input. Recent analyses have underscored that rare cancers present unique interpretive challenges, as many genomic events acquire meaning only when evaluated within tumor-specific biological frameworks [47,48]. This need for expert contextualization is further reinforced by the recent consensus recommendations of the Italian Sarcoma Group, which advocate for extended molecular profiling in mesenchymal tumors and emphasize that translating complex genomic findings into clinically actionable strategies necessitates structured, tumor-type-focused molecular tumor board review [49].Together, these data highlight that MTBs are not only valuable for prioritizing targeted therapies but are essential to ensure accurate, disease-specific interpretation of genomic results in rare and histologically complex malignancies.

Another key challenge is the time-intensive process of identifying relevant evidence [37,50]. Integration of bioinformatics tools and automated platforms may streamline data analysis and improve efficiency [21,36]. Virtual MTBs represent a scalable solution, particularly for resource-limited centers, by improving access to expertise and reducing the need for referrals [7,9,11,17,20,29,35,41,42,45,50]. From the patient perspective, multidisciplinary decision-making may improve trust, satisfaction, and access to clinical trials or targeted therapies [4,6,8,16,19,39,41,45,51,52].

However, a persistent gap exists between identification of actionable alterations and implementation of MTB recommendations, with adherence rates often below 40% [3,11,14,16,25,26,29,31]. Barriers such as limited drug access, restrictive trial eligibility, and financial constraints continue to limit real-world applicability [6,11,14,15,18,25,28].

The genomic landscape analysis highlights the consistent involvement of key driver mutations, including TP53, KRAS, and PIK3CA [4,5,7,8,10,12,14,15,18,24,25,26,29,33], supporting their relevance in precision oncology. Although based on study-level data, these findings align with known molecular patterns in solid tumors. The high rate of actionable alterations (approximately 65–70%) underscores the clinical potential of genomic profiling. However, the gap between detection and implementation emphasizes the need for improved access to therapies, broader clinical trial availability, and stronger infrastructure [3,19,35,37]. Technological integration may further improve efficiency. Automated platforms and bioinformatics tools can support data processing, reporting, and clinical decision-making, although implementation requires significant resources [8,21,36,45,46,53,54,55,56]. Virtual MTBs may provide a cost-effective alternative while expanding access to specialized expertise [9,11,17,18,19,20,26,42,57]. These findings have important clinical implications, including improved physician confidence in genomic testing, enhanced access to expert guidance, and more informed decision-making.

**Figure 2:**
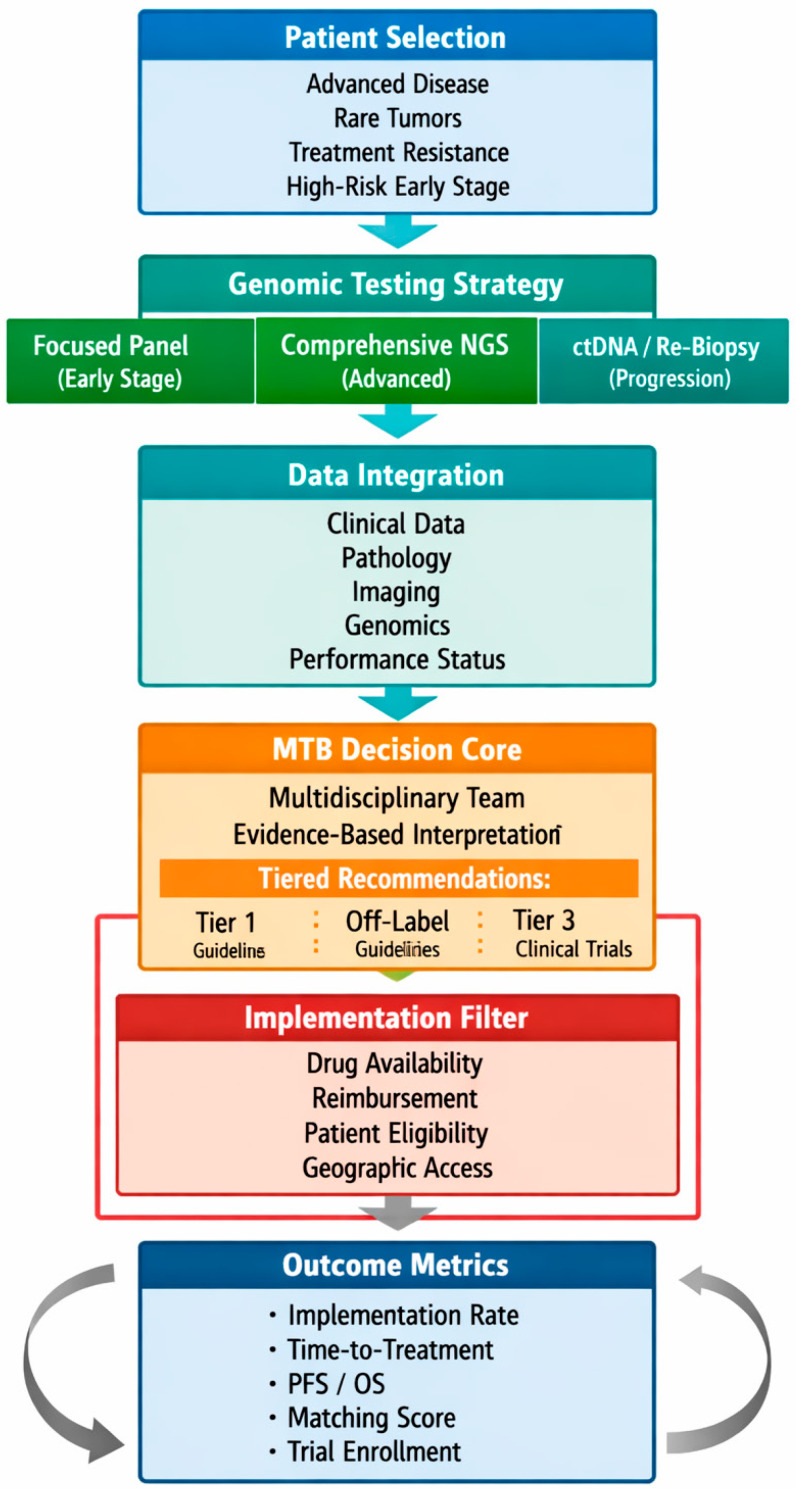
MTB framework proposal.

Figure 2 presents a proposed framework for MTB implementation describing patient selection strategy, genomic testing strategy, data integration, MTB decision core, implementation filter, and outcome metrics to be reported.

A deeper examination of the actionability–implementation gap shows that multiple categories of barriers impede the uptake of MTB recommendations. These factors can be broadly grouped into system-level, physician-level, and patient-level domains. System-level barriers include lack of access to targeted therapies (especially off-label agents), regulatory constraints, insurance or reimbursement limitations, and delays related to sequencing workflows. Physician-level barriers involve differences in clinical judgment, limited familiarity with emerging molecular evidence, and competing therapeutic priorities. Patient-level barriers include rapid clinical deterioration, comorbidities precluding treatment, and individual preference.

Across the included studies, adherence to MTB recommendations remained below 40%, and the reasons for non-adherence clustered consistently into these three domains. The most frequent causes were clinical deterioration before therapy could be initiated, lack of access or reimbursement, and physician-driven treatment decisions diverging from MTB suggestions. Categorizing these findings highlights that the gap between actionability and implementation reflects structural and clinical constraints rather than lack of utility of MTBs per se. A systematic approach to addressing barriers at each level may therefore be necessary to increase the real-world impact of precision oncology MTBs.

Consensus-based approaches may improve treatment appropriateness, increase implementation rates, and support patient involvement, ultimately contributing to better outcomes [3,7,10,14,25,31,51,58,59,60,61,62,63].

AI in the context of MTBs is increasingly recognized as a promising tool for strengthening MTB workflows. AI-based systems may support automated variant classification, literature curation, therapy prioritization, and rapid matching of patients to clinical trials, thereby reducing manual workload and improving consistency in decision-making. More advanced models integrating genomics with clinical and radiologic data have the potential to enhance prediction accuracy and identify treatment strategies that may be overlooked by manual review. At the same time, several limitations must be acknowledged. AI performance is highly dependent on data quality, tumor-type-specific evidence, and robust training cohorts, which remain limited for many rare cancers. Concerns regarding interpretability, reproducibility, regulatory compliance, and potential bias further constrain real-world implementation. As such, AI is likely to function as an adjunct rather than a replacement for multidisciplinary expertise, supporting—but not substituting—the role of MTBs in precision oncology [64,65].

Tools such as MatchMiner, for example, facilitate precision-medicine trial matching by identifying suitable clinical trials for individual patients and highlighting eligible patients for specific studies, thereby accelerating enrollment in precision-oncology trials. Similar digital infrastructures, including classification systems like OncoTree, further improve the consistency of genomic interpretation and trial assignment. These developments, supported by recently published precision-oncology frameworks and consensus recommendations, illustrate how AI can function as a complementary decision-support layer within MTBs, enhancing efficiency while maintaining the need for expert multidisciplinary oversight [64,65].

Limitations: The results should be interpreted considering substantial heterogeneity across studies, including variability in tumor types, patient populations, timing of genomic testing, and comparator definitions. Differences in access to therapies and clinical trials may also influence outcomes. Methodologically, although this study incorporates elements of quantitative synthesis, inconsistent reporting—particularly regarding hazard ratios, confidence intervals, and patient-level data—limited the ability to perform a formal meta-analysis. Therefore, the pooled estimates should be considered exploratory and hypothesis-generating.

Future research should prioritize standardized reporting of MTB outcomes, including uniform definitions of actionable alterations and consistent survival metrics. Prospective studies across diverse healthcare settings, including low-resource environments, are needed to optimize MTB implementation and strengthen evidence in precision oncology.

The role of molecular tumor boards (MTBs) is expected to grow as precision oncology advances. Key future directions include integrating artificial intelligence to streamline genomic interpretation, prioritize therapies, and match patients to clinical trials. The use of multi-omics data—such as transcriptomics, proteomics, and epigenomics—may improve patient stratification and reveal new therapeutic targets. Standardized digital platforms that combine clinical, radiological, and molecular information could support virtual MTBs and enhance data sharing across institutions. Finally, expanding access to targeted therapies and clinical trials through decentralized trial models, improved reimbursement, and international collaboration will be essential for increasing the real-world impact of MTB recommendations.

## 5. Conclusions

Integrating precision oncology into clinical practice represents the present and supports effective interpretation of the genomic landscape, enhances molecular tumor characterization, and broadens access to additional therapeutic targets. This approach also helps establish a clearer clinical pathway, ultimately improving patient outcomes and contributing to survival benefits through the delivery of high-quality cancer care [3,4,6,8,17]. In addition to these advantages, interpreting data generated through molecular profiling demands appropriate expertise and infrastructure, both of which are essential for successfully scaling MTB implementation [21,36,45,47]. Overcoming these limitations may further enhance the establishment of tumor boards, ultimately improving access to state-of-the-art treatment [14,31,38].

Beyond this, relying solely on NGS-based testing marks only an initial step. There is a need for the medical community to work toward fully “democratizing” precision oncology across all stages—from diagnostic testing to molecular tumor boards and therapeutic access—on a global scale. Achieving this requires expanding the availability of genomic and genetic testing, improving access to targeted and immunotherapies, and ensuring broader opportunities for patients to participate in clinical trials.

## Figures and Tables

**Table 3:** Survival benefit following MTB case review for all tumor subtypes in the reviewed articles.

**PFS Benefit**	
Charo et al. [16]	9.3 vs. 3.4 months, HR = 0.5
Kato et al. [22]	6 vs. 4 months (MS > 50) *, HR = 0.63
Louie et al. [33]	6.4 vs. 3 months (MS > 50) *, HR = 0.48
Miller et al. [11]	186 vs. 145 days
Tarawneh et al. [20]	4.3 vs. 1.9 months
**OS benefit**	
Charo et al. [16]	17.1 vs. 10.8 months, HR = 0.64
Hoefflin et al. [18]	18 vs. 8 months
Kato et al. [22]	17 vs. 10 months, HR = 0.67
Louie et al. [33]	15.3 vs. 4.7 months, HR = 0.46

* Matching Score (MS) = matching between tumor alterations and administered therapy.

**Table 4:** Survival benefit following MTB case review for specific tumor subtypes in the reviewed articles.

**Tumor Type**	**PFS Benefit**	
NSCLC *	Koopman et al. [32]	6.3 months (interquartile range 3.2–10.6 months)
CRC **	Louie et al. [27]	3.9 vs. 3.1 months, HR = 0.41
**Tumor type**	**OS benefit**	
Pancreatic cancer	Dorman et al. [32]	24.6 (M0) and 14.1 months (M1) ***
NSCLC *	Huang et al. [17]	HR = 8.15 (when not reviewed by MTB)
NSCLC *	Koopman et al. [32]	10.4 months (interquartile range 6.3–14.6 months)
Tumor type	PFS benefit	
NSCLC *	Koopman et al. [32]	6.3 months (interquartile range 3.2–10.6 months)
CRC **	Louie et al. [27]	3.9 vs. 3.1 months, HR = 0.41

* NSCLC= Non-small-cell lung cancer. ** CRC = Colo-rectal cancer. *** M0 = non-metastatic at diagnosis/M1= metastatic at diagnosis.

**Table 5:** Reports on study type, cancer type, outcomes and notes on heterogeneity among for the exploratory quantitative analysis.

Study	Study Type	Cancer Types	Outcome	MTB vs. Non-MTB	HR (If Reported)	Notes on Heterogeneity
Charo et al. [16]	Retrospective cohort	Multiple solid tumors	PFS	9.3 vs. 3.4 months.	0.5	Mixed tumor types; heterogeneous therapies
			OS	17.1 vs. 10.8 mo.	0.64	Follow-up varies
Kato et al. [22]	Prospective observational (MS > 50 subgroup)	Multiple solid tumors	PFS	6 vs. 4 months.	0.63	Includes high TMB subgroup
			OS	17 vs. 10 months.	0.67	Targeted vs. non-targeted comparison
Louie et al. [33]	Retrospective	Multiple solid tumors (MS > 50 subgroup)	PFS	6.4 vs. 3 months.	0.48	Biomarker-enriched cohort
			OS	15.3 vs. 4.7 months.	0.46	High selection bias possible
Miller et al. [11]	Retrospective	Mixed solid tumors	PFS	186 vs. 145 days	Not reported	Endpoints reported in days; no HR
Tarawneh et al. [20]	Retrospective	Mixed tumors	PFS	4.3 vs. 1.9 months.	Not reported	High variability in treatment lines
Hoefflin et al. [18]	Retrospective	Mainly GI tumors	OS	18 vs. 8 months.	Not reported	Tumor-specific focus, non-HR reporting

## Data Availability

The original contributions presented in this study are included in the article. Further inquiries can be directed to the corresponding author.
